# Identification of heterologous Torque Teno Viruses in humans and swine

**DOI:** 10.1038/srep26655

**Published:** 2016-05-25

**Authors:** Marvin A. Ssemadaali, Karl Effertz, Pankaj Singh, Oleksandr Kolyvushko, Sheela Ramamoorthy

**Affiliations:** 1 Department of Veterinary and Microbiological Sciences, North Dakota State University, Fargo, ND, USA.

## Abstract

Torque Teno Viruses (TTVs) are ubiquitous viruses which are highly prevalent in several mammalian species. Human TTV’s are epidemiologically associated with several human disease conditions such as respiratory illnesses, auto-immune disorders and hepatitis. Recently it was found that swine TTV’s (TTSuVs) can act as primary pathogens. The common occurrence of TTVs as environmental contaminants and the increasing interest in the use of swine organs for xenotransplantation lend importance to the question of whether TTV’s can cross-infect across species. In this study, we examined human and swine sera by swine or human TTV-specific PCRs, to determine whether swine TTVs (TTSuV) DNA can be detected in humans and vice versa. Surprisingly, both human and TTSuV DNA were present in a majority of the samples tested. Transfection of human PBMC’s with TTSuV1 genomic DNA resulted in productive viral infection which was sustained for the three serial passages tested. Lymphoproliferative responses in infected human PBMCs were diminished when compared to the controls. Furthermore, mild to moderate antibody responses against the TTSuV1 ORF2 protein was detected in 16 of the 40 human sera by ELISA. Therefore, these study findings provide initial and fundamental evidence for possible cross-species transmission of TTVs.

Torque teno viruses are small DNA viruses which were discovered as a possible cause of post-transfusion hepatitis in humans^1^. Since then, TTVs have been detected in many mammalian hosts; including dogs, cats, chimpanzees and swine^2^. While the prevalence of TTVs in other species has not been studied extensively, they are reported to range from 5–90% in humans^3^,^4^, and about 55–100% in swine^5^,^6^. The virus is detected in all major organs, secretions, excretions, blood and blood cells. The tissue distribution and localization of TTVs are similar in humans and swine^3^,^7^.

Generally, TTVs establish chronic infections without causing overt pathology. Hence their role as primary pathogens is a subject of scientific debate. Several epidemiological studies have associated TTVs with a spectrum human diseases such as hepatitis B or C, multiple sclerosis, hepatocellular carcinomas, respiratory infections, blood disorders and autoimmune diseases^8^,^9^. In swine, experimental infection of gnotobiotic pigs with swine Torque Teno virus 1 or 2 (TTSuV1 or 2) causes mild to moderate respiratory, hepatic and nephritic lesions, indicating that TTSuVs can act as a primary pathogens in swine. In experimental coinfections, TTSuV’s potentiated other swine viral diseases^10^,^11^. Therefore, the question of whether TTVs can establish cross-species infections is of considerable importance.

The lack of a reliable cell culture system has limited the exploration of the molecular biology and pathogenesis of TTVs. However, recent studies show that TTV proteins encode auto-reactive epitopes which are also detected in multiple sclerosis and lupus patients^12^, and that a TTV encoded miRNA depresses host interferon signaling^13^. Viremia in TTV-infected individuals is inversely correlated with immune status. Indeed, it has been suggested that TTV DNA loads can be used as an indicator of immuno-suppression^14^,^15^. Therefore, in immuno-compromised individuals, the possibility that TTVs could replicate to high levels and facilitate pathology cannot be ruled out.

Widespread environmental contamination, based on the detection of human TTV (huTTV) DNA, is extremely common in water sources^16^,^17^,^18^, sewage^19^ and in air or on surfaces, especially in hospitals^20^. Contamination of swine-derived laboratory enzymes such as trypsin and some veterinary vaccines with TTSuVs is also reported^21^. Current screening protocols for blood donors do not include detection of TTVs. However, given their ubiquitous nature, TTVs are also potential contaminants of the blood supply^22^. Humans are likely to frequently ingest TTSuVs in food and water. Both pork products and human feces contain TTSuV DNA^23^,^24^. Moreover, with the availability of improved technology, there is an increased interest and potential for the use of swine-based xenotransplantation products^25^. Therefore, from a public health perspective, it is especially critical to determine whether TTSuVs can establish infections in humans.

In this study, we examined sera from humans and swine for the presence of TTSuV and human TTVs (huTTV) DNA by PCR. Interestingly, both TTSuV and huTTV DNA were detected at high levels in both species. We also determined that TTSuV1 can serially infect human PBMCs and reduce their ability to proliferate in response to mitogens. Antibody responses to TTSuV1 were detected in some human samples, indicating that TTSuVs can potentially establish infections in humans. Our data provides key, primary evidence for the possible transmission of TTVs between mammalian species and is significant in understanding the ecology and pathogenesis of this highly-prevalent virus.

## Results

### Detection of TTSuV DNA in the human and swine sera

To determine whether TTSuV DNA can be detected in human sera and vice versa, we examined a total of 60 sera samples. Forty of the samples were from healthy humans while 20 were from a high-health, swine herd. All sera were examined by 4 PCR’s targeting various regions of the TTSuV genome (Table 1) and a human TTV-specific PCR. Detection of TTSuV1 and 2 DNA by the real-time quantitative pan-TTV PCR, showed that 17 of the 20 swine samples tested were positive. Surprisingly, 32 of the 40 human samples tested were positive for TTSuV DNA by this assay (Table 2). To further ensure the accuracy of our results, the samples were tested by a previously validated gel-based PCR (TTSuV1 UTR2)^5^ which targeted a different region of the conserved UTR. Twenty-seven of the 40 human samples also tested positive on the second PCR confirming the presence of TTSuV1 DNA in the human sera. Twelve of the 20 swine samples tested positive. To further validate our results, the samples were tested with PCRs targeting the more variable protein coding regions for TTSuV1 ORF2 and 3. With the TTSuV1 ORF2-specific PCR, 8 of the 40 human samples and 6 of the 20 swine samples were positive. Two of the human samples and 11 of the swine samples were positive by the TTSuV1 ORF3-specific PCR (Table 2). Swine TTV sequences, specific to the UTR, ORF2 or 3 respectively were returned when the sequences obtained from the PCR products were subjected to a nucleotide BLAST analysis (Table 1), thus validating the specificity of the assays. All no-template controls were negative in each PCR run.

### Detection of huTTV DNA in the human and swine sera

To detect the presence of hu TTV DNA, the human and swine sera were examined by a pan human TTV-specific PCR (Table 1). As expected, 85% of the human sera were positive for huTTV DNA. A nucleotide BLAST analysis of sequenced amplicons returned accession numbers KJ082064.1 and AY590626.1, both of which were huTTV sequences from China and Venezuela respectively. Of the 20 swine sera, 16 tested positive for huTTV DNA (Table 2). Accession numbers AB041007 and AF122915, consisting of huTTV sequences, were the top hits for the sequenced amplicons (Table 1). The human and swine sequences obtained were genetically distinct. In general, swine and human TTV genomes share less than 50% sequence identity^26^. All no-template controls were negative in each PCR run.

### Infectivity of TTSuV1 for human PBMCs

**Figure 1:**
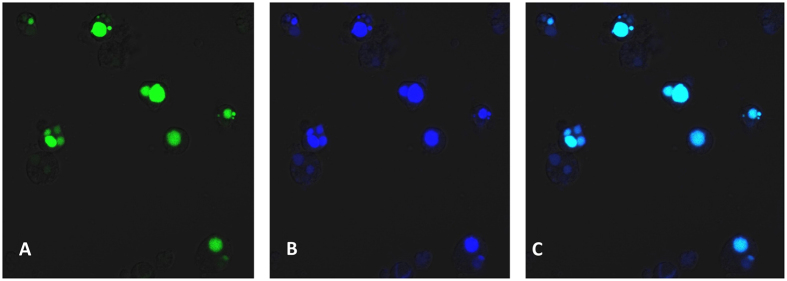
Indirect Immuno-Fluorescence Assay [IFA] detection of TTSuV1 replication in human PBMCs. Recombinant TTSuV1culture, rescued by transfection of human PBMCs with the circularized viral genome, was passaged three times in human PBMCs. A representative image from the third passage is depicted. (**A**)–Apple green, nuclear fluorescence in human PBMCs infected with TTSuV1 and stained with an anti-TTSuV1 ORF1- specific antibody, (**B**)–Blue, nuclear counter-staining with DAPI, (**C**)–Overlay image of A and B showing the nuclear localization of the replicating TTSuV1. Untransfected negative controls did not show specific fluorescence [image not shown].

To determine whether a TTSuV1 can produce sustained infections of human immune cells, virus culture derived by transfection of the TTsuV1 genome in human was serially passaged three times in human PBMCs Bright green, nuclear fluorescence indicative of TTSuV1 replication was evident in all the three serial passages tested. The negative controls did not show TTSuV1-specific staining but virus-specific nuclear staining was detected in both monocytes and lymphocytes, indicating that the cell types present in PBMCs support TTV replication and that TTSuVs can replicate in human PBMCs (Fig. 1). Prior to transfection, the PBMC’s were negative when assessed by the pan human and TTSuV PCRs.

### Lymphocyte proliferation responses in infected immune cells

**Figure 2:**
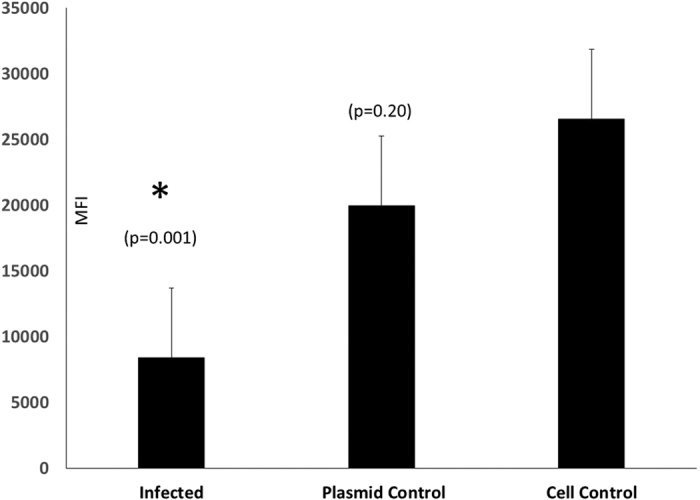
Ability of TTSuV1 infected human PBMCs to proliferate in response to mitogens. Five replicates each of human PBMC’s were either infected with TTSuV1, an unrelated DNA control or remained as uninfected cell controls. Bars represent the average mean fluorescence intensity [MFI] values of the five replicates after subtraction of the mean background fluorescence of the media controls. *Significantly different from the cell control as assessed by a Student’s T test.

To determine whether the infection of human PBMCs with TTSuVs can affect immune function, the ability of the infected cells to respond to non-specific stimulation by a mitogen was assessed using a non-radioactive, dye based assay^27^. When stimulated with PHA, the mean fluorescence intensity (MFI) of infected cells was lower than that of the uninfected control cells, indicating that TTSuV1 infection diminished the capacity of the PBMCs to respond to immune stimulation (Fig. 2). The difference was statistically significant with p = 0.001. The differences in the proliferative capacity of transfected with an unrelated DNA and was not statistically significant in comparison to the uninfected control cells, although slightly reduced. The difference between the cells infected with TTSuV1 and cells transfected with the unrelated DNA was highly significant at p = 0.0002 (Fig. 2).

### Antibody responses to TTSuV1 ORF2

**Figure 3:**
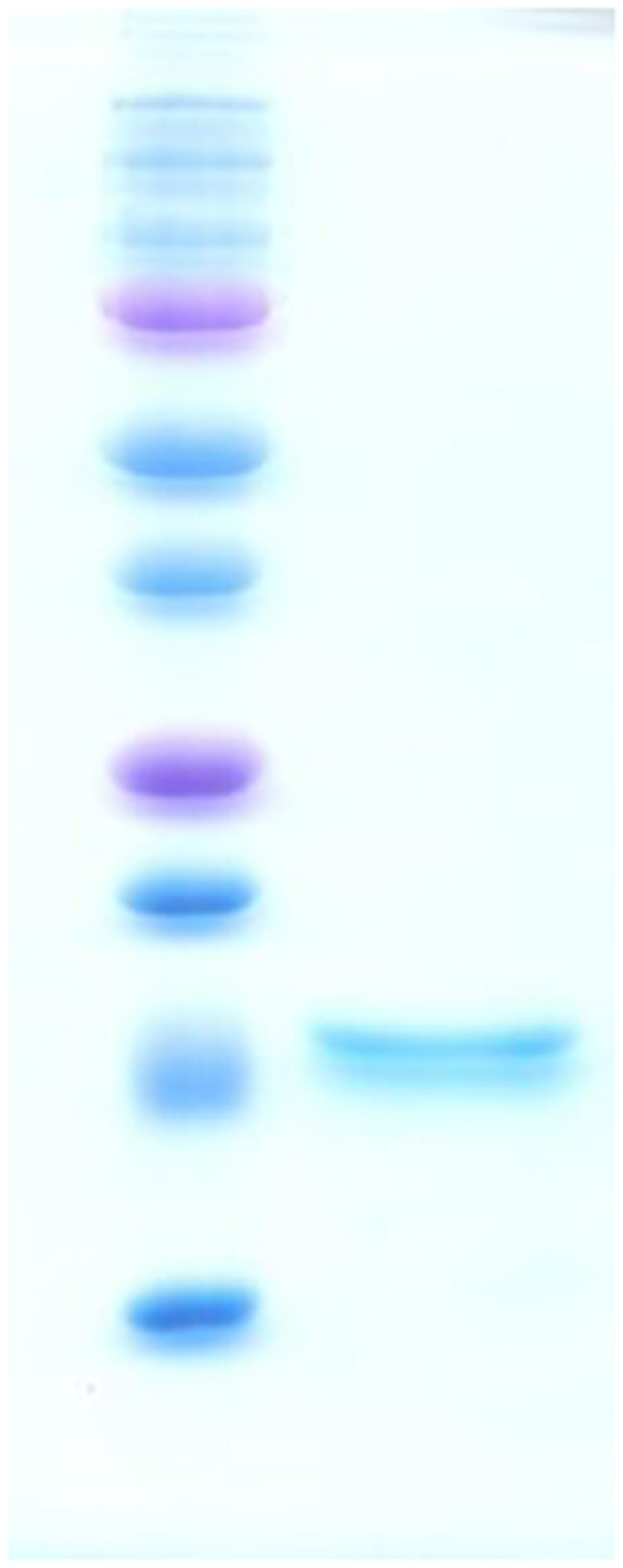
Expression of the TTSuV1 ORF2. TTSuV1 ORF2 protein purified by HIS tag affinity purification from lysates of transformed *E.coli* BL21 DE3 cells. Coomassie blue stained, SDS-PAGE gel showing the protein ladder in the left lane and purified TTSuV1-ORF2 [approximately 10 kDa] in the right lane.

To prepare capture antigen for the detection of TTSuV1-specific antibodies, the TTSuV1 ORF2 protein was expressed in a bacterial expression system. The recombinant protein was obtained in a soluble form at the expected molecular weight of approximately 10 kDa. Affinity purification led to the separation of a single protein, as determined by SDS-PAGE analysis (Fig. 3). The purified protein also showed specific reactivity to a commercial anti-HIS tag antibody by Western blotting and rabbit hyper-immune anti-ORF2 antibody (data not shown).

**Figure 4:**
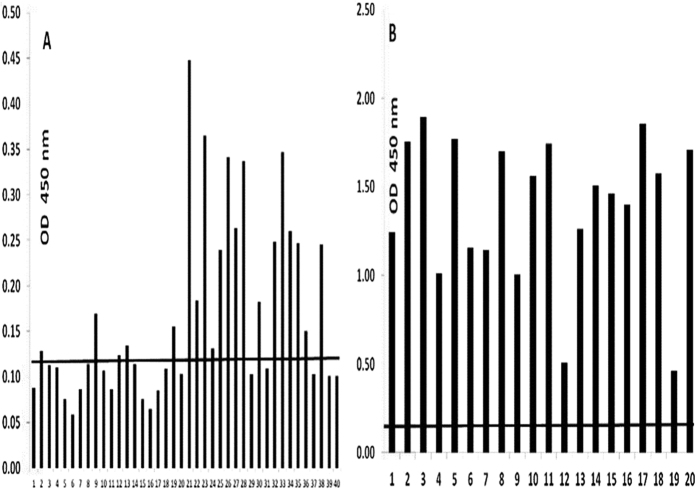
(**A,B**) ELISA for the detection of antibody responses to TTSuV1: Fig. 4A–Antibody responses to the TTSuV1 ORF2 protein in human sera (N = 40), Fig. 4B–Antibody responses to the TTSuV1 ORF2 protein in swine sera (N = 20). The mean optical density values of duplicate values are depicted. The cutoff value for the human sera (0.125) was calculated as the lowest quartile value of the data set +/− two standard deviations. The cutoff value for the swine samples (0.16) is the value of a negative control sample which was obtained after screening a panel of field swine sera.

To determine whether active TTSuV1 infection indicated by sero-conversion occurs in healthy individuals who are PCR positive, the human and swine sera were examined by a TTSuV1-specific ELISA, using the purified TTSuV1 ORF2 protein as the capture antigen. Low to moderate (OD values ranging from approximately 0.15 to 0.45) antibody responses, which were above the cutoff value of 0.125 (calculated as the lowest quartile value +/− two standard deviations) were detected in 16 of the 40 human sera tested, indicating that sero-conversion to TTSuV occurred in human beings. Among the swine samples tested, except for two animals with low titers, all other animals had high antibody levels against the TTSuV1-ORF2 protein (Fig. 4), indicating that that antibody responses to the ORF2 protein was mounted in natural infections of swine. All ELISA-positive samples were also TTSuV PCR positive except for swine sample 18.

## Discussion

With the recently published evidence for the pathogenicity of TTSuVs in swine^10^,^11^, the detection of TTSuVs in pork products^24^, human drugs and veterinary vaccines^21^ and its ubiquitous presence in the environment^16^,^20^, understanding whether TTSuVs can infect human beings and whether TTVs, in general, can cross species barriers has become both important and essential.

High levels of genetic diversity is common in TTV’s^28^.The detection huTTV DNA in 85% of the human sera tested in this study is consistent with prevalence estimates of between 5–90% in other studies^3^,^4^, depending on the sensitivity of the assay used. Unexpectedly, in this study, only 3 out of the 40 human samples tested negative for TTSuV DNA on all the assays used (Table 2). To rule out a sample-source bias, samples were purchased from two different vendors, while ensuring that all samples were from individual donors. To ensure validity, samples were also tested with PCRs targeting four different regions of the TTSuV genome. The pan huTTV^29^ and pan TTSuV real-time PCR assays used were highly sensitive, with a detection limit of 1.5 copies per reaction, which is comparable to other qPCRs for the detection of TTVs^30^. As previously described in detail^5^, the gel-based PCR for the detection of TTSuV1 was less sensitive at 1000 copies per reaction. Overall, detection was consistent between two assays, considering that TTSuV2 was not detected by the gel based assay, the sensitivity was lower in the gel based assay and that different regions of the UTR were targeted by the two assays. The amplicons of the two PCR’s targeting the TTSuV UTR’s were highly conserved; with an average percentage similarity of over 90% when aligned with other TTsuV1 and 2 or TTSuV1. The finding that fewer samples were positive with PCR’s targeting the TTSuV1 ORF2 and ORF3 is consistent with the fact that they are more variable. Human fecal matter was previously shown to contain TTSuVs DNA^23^, and its presence can be attributed to the contamination of water supplies with swine manure^19^. It is likely that environmental or feed contamination, rather than proximity to swine, plays a significant role in the exposure to TTSuVs in humans, plays a significant role in the exposure to TTSuVs in humans. While our findings call for a more extensive characterization of the animal to human transmission of TTSuV’s involving a larger sample size, sequential sampling and incorporating pork consumption as a variable, this study provides important initial evidence for the potential zoonosis of TTSuV’s.

Currently, TTV’s are classified in a species-specific manner, with this report being the first to present data supporting a possible cross-species transmission. Although the genomes of TTV’s from various animal species, including humans, are similarly organized; each species-specific TTV is genetically distinct. They differ from each other and human TTV’s by more than 50% sequence identity^26^. Therefore, the data presented does not warrant a reclassification of TTVs.

Of the 20 swine samples examined in this study, only one was negative for TTSuVs. The high prevalence of TTSuVs in this sample set compared to our previously published rate of approximately 55%^5^ is likely due to the fact that the samples were collected from multiparous, adult sows, while the population estimation was carried out in production animals which were likely to be younger^6^. Surprisingly, despite being derived from a “closed” herd with no exposure to outside animals, a majority of the swine samples in our study were positive for huTTV DNA (Table 2). While exact estimates of the extent of contamination of water sources within the U.S with huTTV DNA are not available, extensive contamination has been reported in other parts of the world^16^,^18^. Moreover, huTTV DNA has been previously detected in buffalo milk^31^.

The presence of DNA in fecal matter could represent a merely transient passage through a non-definitive host. However, the presence of TTSuV DNA in human serum could be indicative of viremia. Since recent findings support a possible role for TTSuVs as the primary or secondary etiological agents of viral infections in swine^10^,^11^ the question of whether TTSuVs can replicate in humans is important from a public health perspective. While the exact cell types which support TTV replication have not been identified, it has been suggested that T lymphocytes and PBMCs can support replication^32^. Our findings that human PBMCs are able to support serial infections with TTSuV1, not only confirm that PBMCs can support viral replication, but also show that PBMCs are a possible site of viral replication in cross-species TTV infections. Additionally, our finding that the ability to respond to immune stimuli is diminished in TTSuV1-infected PBMCs provides preliminary evidence that TTVs can be immuno-suppressive.

Widespread detection of antibody responses to TTSuVs in swine and huTTVs in humans has been previously described^33^,^34^. While antigenic cross-reactivity between human and swine TTVs has not been studied extensively, a human genogroup 1-specific anti-serum did not cross-react with the TTSuV1 ORF1 antigen^33^. The TTSuV1 ORF2 protein is about 53% similar between the TTSuV1a and 1b subtypes, while it is about 40% similar to the TTSuV2 ORF2 proteins, and about 20% similar to its counter-part in human TTVs. Therefore, the detection of antibody responses to the TTSuV1 ORF2 protein in 16 of the 40 human sera tested further substantiates our hypothesis that TTSuV’s can be potentially zoonotic. While exact information about when the individuals in this study were infected is not available, patterns of sero-conversions and sero-reversions, where PCR-positive individuals lose detectable serum antibodies in subsequent samplings, have been detected in TTV-infected individuals^35^. Similarly, in infections of swine with the closely related porcine circoviruses, we have detected a waxing and waning pattern of viremia, probably in response to host immunity, especially in older animals in which chronic infection has been established^36^. Therefore, a combination of PCR and antibody detection is likely to provide accurate evidence for active viremia as well as previous exposure for TTVs.

In conclusion, our findings are the first to support the possibility that TTV infections can be zoonotic or reverse zoonotic. With the abundance of epidemiological data linking TTVs to various human diseases, the possibility of opportunistic pathogenicity cannot be ignored. While healthy humans will most likely be able to clear TTSuV1 infection, more detailed studies are required to determine if TTSuVs or TTVs from other mammalian species can establish infections or alter immune functions in immuno-compromised individuals, especially because the inverse correlation between TTV viral loads and the immune status of the individual is well-established^14^,^15^. Further research is required to determine the significance of these initial findings in the context of host immunity or pathology and is the focus of our future research.

## Materials and Methods

### Serum Samples

A total of 60 sera, comprising human (N = 40) and swine (N = 20) were tested by swine or human TTV-specific PCRs as indicated in Table 1. Swine samples were obtained from a herd maintained as a source of experimental animals for university research. The human sera purchased were collected with informed consent and the approval of the institutional review boards of two different commercial vendors (Valley Biomedical, Winchester, VA or Bioreclamation IVT, Long Island, NY). All human samples were screened to be negative for HBsAG, HIV 1/2 Ab, HCV Ab, HIV-1 RNA, HCV RNA and STS by the vendor. The end users were blinded to the identity of the donors of the samples. All experimentation was carried out with the approval of the N. Dakota State University’s institutional biosafety committee and in accordance with the approved guidelines.

### PCR Detection of Human TTV DNA

For the detection of human TTV DNA, a previously described pan-human TTV (huTTV) PCR^29^ was adapted to a real-time PCR format. Briefly, DNA was extracted from the sera using the QiaAmp DNA Mini kit (Qiagen, Valencia, CA). Primers (Table 1) were added to the iTaq™ Universal SYBR^®^ Green Supermix (Biorad, Hercules, CA), with 25 ng of template DNA, cycled 40 times (iCycler CFX96 Touch Real Time PCR Detection system, Biorad, Hercules, CA) at a Tm of 60 °C.

### PCR Detection of TTSuV DNA

To ensure reliable detection, four different PCRs (Pan TTSuV UTR PCR 1, TTSuV1 UTR PCR2, TTSuV1 ORF2, and TTSuV1 ORF3-Table 1) were used for the detection of TTSuV DNA in the human and swine sera. Two PCR’s designated as the pan-TTSuV UTR PCR 1 and TTSuV1 UTR PCR2 targeted two non-overlapping regions of the conserved untranslated region (UTR). The pan-TTSuV PCR1 detected both the TTSuV1 and 2 genotypes while the TTSuV1 UTR2 targeted the UTR of the more commonly prevalent TTSuV1 genotype^5^. Two other PCRs (TTSuV1 ORF2 & 3) targeted the more variable ORF2 and 3 regions of the TTSuV1 genotype (Table 1).

The pan-TTSuV PCR1 was carried out essentially as described^6^ except that iTaq™ Universal SYBR^®^ Green Supermix (Biorad, Hercules, CA), with 25 ng of template DNA, primers, a Tm of 57 °C and 36 amplification cycles was used. The TTSuV1 PCR 2 was carried out as previously described^5^, except that the TTSuV2 primers were not used. The PCR’s targeting the TTSuV1 ORF1 and 2 were carried out using the primers listed in Table 1, 25 ng of template DNA in a commercial PCR master mix (ReadyMix^™^ Taq PCR Reaction Mix, Sigma), and a Tm of 56 °C for 35 cycles.

For all PCR’s, two no template controls were included and samples were tested in duplicate or nested. The specificity of all PCR assays was determined by a nucleotide BLAST analysis of two sequenced amplicons from each species.

### Genome cloning and sequencing

The TTSuV1 genome was amplified from the bone marrow of a swine diagnostic case. Two opposing primers (5′-GACAATTAATTTATGCAAAGTAGGA-3′ and 5′-GACAATTAA TTTGCATAAACTCCGC-3′) with flanking Ase-I sites were used to amplify the entire circular genome, which was then cloned into the pCR2.1 TA cloning vector (Invitrogen, Carlsbad, CA). The genome was sequenced and deposited in GenBank (KT037083).

### Permissiveness of human PBMCs to TTSuV1

To ensure they were PCR negative, human peripheral blood mononuclear lymphocytes (PBMCs) were tested by the pan human and TTSuV PCR’s as described above. Cells were cultured at 5 × 10^5^ cells/ ml in 10% DMEM with 8 μg/ml of anti-CD3 antibody (Tonbo Biosciences, San Diego, CA), 1 μg/ml of phytohemagglutinin (PHA-M) (Life Technologies, Grand Island, NY, USA) and human rIL-2 (10 U/ml) (Tonbo Biosciences) for 48 hours. The 2877 bp genome of TTSuV1 was excised from the shuttle vector by Ase-I digestion, purified by gel extraction and re-circularized by ligation (T4 DNA ligase, New England Biolabs, Ipswich, MA). The cultured PBMCs were transfected with the circularized genome at 1 ug/well TransIT-2020 (Mirus Bio, Madison, WI), following the manufacturer’s instructions. One replicate of cells was maintained as the untransfected negative control. Plates were incubated for 48 hours at 37 °C in a CO_2_ incubator. The rescued virus culture was used to infect new human PBMC cultures, as described above and serially passaged three times.

Viral replication was assessed using an indirect immunofluorescence assay (IFA). Briefly, the adherent PBMCs and non-adherent PBMC’s were washed in Hanks Balanced Salt Solution (HBSS) (Mediatech/Corning, Manassas, VA) and fixed in ice cold, acetone: methanol (1:1). Fixed cells were stained with a rabbit polyclonal anti-ORF1 TTSuV1b antibody^37^ at a 1:100 dilution for 2 hrs at 37 °C, followed by a 1:50 dilution of anti-rabbit FITC (KPL, Gaithersburg, MD) conjugate for 45 mins. Stained slides were from each serial passage examined by confocal microscopy (Zeiss Laser scanning confocal microscope, NDSU Core imaging facility).

### Effect of TTSuV1 infection on human PBMC proliferation

Five replicates each of plated PBMCs were either transfected, as described above, with the circularized TTSuV1 genome, an unrelated linearized plasmid DNA (pcDNA™ 3.1/V5-His TOPO^®^, Life Technologies, Grand Island, NY) or served as untransfected negative controls, while growth media alone was used to control for background fluorescence. All cells were stimulated 1 μg/ml of phytohemagglutinin (PHA-M) (Life Technologies, Grand Island, NY) for 48 hours. Lymphocyte proliferation in response to the mitogen stimulation was measured using the Alamar Blue reagent (AbD Serotec/Bio-Rad, Raleigh, NC), according to the manufacturer’s instructions. The data obtained was analyzed by a Student’s T test to determine statistical significance.

### Expression and purification of HIS-tagged TTSuV-ORF2 protein

The TTV-ORF-2 gene was amplified from cloned TTSuV1 genome and shuttled into the pET-28a bacterial expression vector (EMD, Millipore, Billerica, MA) in conjunction with a 6X HIS tag at both the N and C termini. Primers 5′-GTCAAGCTTTGCCGGAACACTGGGAGGAAG-3′ and 5′-ACGT CTCGAGCCAGCCATCGTCGCCGATAGTC-3′ with HindIII and XhoI restriction sites respectively, were used for amplification. The plasmid was transformed into a bacterial expression vector (BL21 DE3, Life Technologies, Grand Island, NY) and induced over-night by the addition of 1 mM IPTG at 37 °C. The over-expressed ORF2 protein was purified by affinity chromatography (His-Spin Protein Miniprep kit, Zymo Research, Irvine, CA), following the manufacturer’s instructions.

### ELISA for the detection of anti-TTSuV antibodies

Plates (High Bind Microplate, Corning^®^, Corning, NY) were coated with 50 μl of a 1:100,000 dilution of the purified TTSuV1 ORF2 the antigen. Coated plates were blocked with 2% BSA and 2% normal sheep serum in a commercial block (General block, ImmunoChemistry Technologies, Bloomington, MN) for 2 hrs at 37 °C, incubated with 1:50 of the human or swine sera for 2 hours at 37 °C, in duplicate, followed by the respective anti-species HRPO-conjugate (KPL, Gaithersburg, MD), at a 1:5000 dilution for 45 mins at 37 °C, and incubation with the substrate (TMB, KPL, Gaithersburg, MD). The reaction was stopped with a 1 M HCl solution after 2 mins. Plates were read at 450 nm on an ELISA plate reader (Elx800 reader, BioTek Instruments, Inc., Winooski, VT).

## Additional Information

**How to cite this article**: Ssemadaali, M. A. *et al.* Identification of heterologous Torque Teno Viruses in humans and swine. *Sci. Rep.* **6**, 26655; doi: 10.1038/srep26655 (2016).

## Figures and Tables

**Table 1:** Human or swine TTV- specific PCR assays used for the assessment of the human and swine sera.

PCR	PCR Type	Target	Forward Primer	Reverse Primer	Size (bp)	BLASTresults
Pan-Human TTV	qPCR	Untranslated region (Conserved)	5′gtaagtgcacttccgaatggctgag3′	5′gcccgaattgcccttgac3′	132	aAB041007.1
						aAF122915.1
						bKJ082064.1
						bAY590626.1
Pan-TTSuV UTR 1	qPCR	Untranslated region (Conserved)	5′cgaatggctgagtttatgcc3′	5′gataggccccttgactccg3′	95	aKR054745.1
						a,bKR131718.1
						bHM633251.1
						bKR054745.1
TTSuV1- UTR 2	Gel-based	Untranslated region (Conserved)	5′gcggtcaaaatggcggaag3′	5′ggacttgagctcccgaccaa3′	124	aEU564163.1
						aJF451574.1
						bEU006509.1
						bJX872390.1
TTSuV1-ORF2	Gel-based	ORF2 (Variable)	5′agtcaagcttttgccggaacactgggaggaag3′	5′acgtctcgagccagccatcgtcgccgat3′	235	aJX535326.1
						aHM633254.1
						bHM633254.1
						bJX535326.1
TTSuV1-ORF3	Gel-based	ORF3 (Variable)	5′gcgacgatggctgtttggaggtgaaataccaaccc3′	5′acgtctcgaggcgtttcttttgttttttat3′	477	aHM633244.1
						aHM633254.1
						bHM633254.1
						bJX535326.1

^a^Top 2 nucleotide BLAST results obtained from the sequenced PCR amplicons two swine samples.

^b^Top 2 nucleotide BLAST hits obtained from the sequenced PCR amplicons of two human samples.

**Table 2:** PCR detection of human and swine TTVs in human and swine serum.

Human Sera					
Sample No	HuTTV-PCR	TTSuV-UTR1	TTSuV1-UTR2	TTSuV1-ORF2	TTSuV1-ORF3
1	+		+		
2	+		+		
3	+	+			
4	+		+		
5	+	+			
6	+	+	+		
7	+				
8	+	+	+	+	+
9	+	+	+		
10	+	+		+	
11	+				
12	+	+	+		
13	+	+	+	+	+
14	+	+	+		
15	+	+	+		
16	+	+	+	+	
17	+	+			
18	+	+	+		
19	+	+	+		
20	+	+	+		
21		+	+	+	
22		+	+		
23		+			
24		+			
25	+	+	+		
26	+		+		
27	+	+			
28	+	+			
29	+	+			
30		+	+		
31	+	+			
32	+	+	+	+	
33	+	+	+	+	
34	+	+	+		
35	+	+	+	+	
36	+	+	+		
37	+	+	+		
38	+		+		
39		+	+		
40	+				
Swine Sera					
1	+	+		+	+
2	+		+	+	
3	+	+			
4	+	+			
5	+	+			
6	+	+	+		
7	+	+	+		+
8	+	+	+		+
9	+	+	+		+
10	+	+	+		+
11	+		+		
12		+	+		+
13	+	+	+		+
14	+	+		+	
15		+		+	+
16		+	+		+
17		+	+	+	
18	+				
19	+	+	+	+	+
20	+	+			+
